# Outcomes and relevance of emergency percutaneous coronary angiography and intervention after resuscitated cardiac arrest: a retrospective study

**DOI:** 10.1186/s12872-024-04052-1

**Published:** 2024-08-13

**Authors:** Baudouin Bourlond, Marion Dupré, Pierre-Nicolas Carron, Lucas Liaudet, Eric Eeckhout

**Affiliations:** 1 Service of Cardiology, Lausanne University Hospital and University of Lausanne, Lausanne, Switzerland; 2 Service of Adult Intensive Care Medicine, Lausanne University Hospital and University of Lausanne, Lausanne, Switzerland; 3 Emergency Department, Lausanne University Hospital and University of Lausanne, Lausanne, Switzerland

**Keywords:** Cardiac arrest, Coronary angiography, Percutaneous intervention, Survival, ST segment elevation

## Abstract

**Background:**

In patients resuscitated from cardiac arrest and displaying no ST-segment elevation on initial electrocardiogram (ECG), recent randomized trials indicated no benefits from early coronary angiography. How the results of such randomized studies apply to a real-world clinical context remains to be established.

**Methods:**

We retrospectively analyzed a clinical database including all patients 18 yo or older admitted to our tertiary University Hospital from January 2017 to August 2020 after successful resuscitation of out-of-Hospital (OHCA) or In-Hospital (IHCA) cardiac arrest of presumed cardiac origin, and undergoing immediate coronary angiography, regardless of the initial rhythm and post-resuscitation ECG. The primary outcome of the study was survival at day 90 after cardiac arrest. Demographic data, characteristics of cardiac arrest, duration of resuscitation, laboratory values at admission, angiographic data and revascularization status were collected. Comparisons were performed according to the initial ECG (ST-segment elevation or not), and between survivors and non-survivors. Variables associated with the primary outcome were evaluated by univariate and multivariate regression analyses.

**Results:**

We analyzed 147 patients (130 OHCA and 17 IHCA), including 67 with STEMI and 80 without STEMI (No STEMI). Immediate revascularization was performed in 65/67 (97%) STEMI and 15/80 (19%) no STEMI. Day 90 survival was significantly higher in STEMI (48/67, 72%) than no STEMI (44/80, 55%). In the latter patients, survival was not influenced by the revascularization status. In univariate and multivariate analyses, lower age, a shockable rhythm, shorter durations of no flow and low flow, and a lower initial blood lactate were associated with survival in both STEMI and no STEMI. In contrast, metabolic abnormalities, including lower initial plasma sodium and higher potassium were significantly associated with mortality only in the subgroup of no STEMI patients.

**Conclusions:**

Our results, obtained in a real-world clinical setting, indicate that an immediate coronary angiography is not associated with any survival advantage in patients resuscitated from cardiac arrest of presumed cardiac etiology without ST-segment elevation on initial ECG. Furthermore, we found that some early metabolic abnormalities may be associated with mortality in this population, which should deserve further investigation.

**Supplementary Information:**

The online version contains supplementary material available at 10.1186/s12872-024-04052-1.

## Background

Out-of-hospital cardiac arrest (OHCA) is associated with a mortality of up to 65% despite adequate resuscitation [1]. The majority of OHCA from presumed cardiac etiology is related to coronary artery disease (60–80% of cases) [2]. In this respect, it is currently recommended that patients resuscitated from OHCA with ST-segment elevation on post-resuscitation ECG should undergo immediate coronary angiography and percutaneous coronary intervention, since a large majority of these patients display coronary artery disease and therefore benefit from early revascularization [2, 3].

In contrast, the role of coronary angiography and PCI in OHCA survivors without ST-segment elevation has been a matter of controversy. Whereas some observational studies [4–6] and a 2020 meta-analysis [7] indicated an improved survival after early PCI in patients resuscitated from OHCA without STEMI, two large recent randomized clinical trials (RCTs) [1, 8, 9] and an updated meta-analysis [10] did not confirm such improvement. Although these latter RCTs do not support an immediate invasive strategy in the absence of STEMI after OHCA, approximately 30% of patients without evidence of STEMI display actual coronary artery disease with an occluded culprit vessel in about two thirds and could therefore benefit from early PCI [5]. On the other hand, performing an immediate coronary angiography may expose these patients to procedural complications, and could result in delays in the search for alternative diagnosis and in the provision of post-resuscitation neuroprotective care, with possible negative impact on the prognosis [1].

To further address these issues in a real-world clinical context, we retrospectively evaluated the impact on survival of early coronary angiography after cardiac arrest in a broad cohort comprising both patients with and without STEMI. Indeed, until 2020, all patients admitted to our tertiary hospital after cardiac arrest of presumed cardiac origin and with sustained return of spontaneous circulation (ROSC), entered a clinical care pathway including an immediate coronary angiography, independently from the post-resuscitation ECG changes. The key clinical questions in our study were (1) to compare the characteristics and outcome of CA between patients presenting with or without STEMI on their initial ECG, (2) to assess the prevalence of coronary artery disease in CA patients without STEMI, and (3) to evaluate the potential benefit from early revascularization in these patients.

## Methods

### Study design and population

Our study was a retrospective analysis of a clinical database including all consecutive patients 18 yo or older admitted to our tertiary University Hospital from January 2017 to August 2020 after successful resuscitation of non-traumatic OHCA or In-Hospital Cardiac Arrest (IHCA) of presumed cardiac origin, and undergoing immediate coronary angiography (performed as soon as possible after hospital admission, and within 120 min from ROSC), regardless of the initial rhythm (shockable or not) and the immediate post-resuscitation ECG, in accordance with an internal clinical practice guideline (“ROSC clinical care pathway”). Following coronary angiography, all patients were transferred to our multidisciplinary 35 bed ICU and treated according to standard protocols of post-cardiac arrest care, notably including targeted temperature monitoring. The study was approved by our local ethical committee (Commission cantonale d’éthique de la recherche sur l’être humain, CER-VD, project number 2019 − 00883). Surviving patients were contacted after the index hospitalization to obtain informed consent. For non-surviving patients, a waiver of consent was authorized. Our study conforms to the STROBE criteria for the reporting of observational studies [11].

### Data collection

The primary outcome of the study was survival at day 90 after cardiac arrest. Demographic variables included age, gender, BMI, cardiovascular co-morbidities, as well as history of ischemic cardiomyopathy and previous coronary interventions. Characteristics of the cardiac arrest were recorded, including location (OHCA vs. IHCA), initial rhythm, witnessed arrest, presence of a defibrillator (internal or external), and the durations of no flow, time to ROSC and low flow. Laboratory data on admission comprised blood leukocytes, serum creatinine, blood urea nitrogen, potassium, sodium, lactate, glucose, high sensitivity Troponin T (HS TnT), creatine kinase and its MB fraction (CK-MB). The admission ECGs were all reviewed by two cardiologists (BB and MD) and the following changes were characterized: ST-segment elevation, ST-segment depression, T wave inversion and Left Bundle Branch Block. According to the initial ECG, patients were categorized as either “STEMI” or “no STEMI” patients. “No STEMI” patients therefore included all patients without ST segment elevation, independently from the etiology of CA. All angiograms were reviewed by two experienced interventional cardiologists (MD and EE) and all revascularization procedures were recorded (including PCI and urgent coronary artery bypass grafting). Coronary artery disease was defined by the presence of a significant stenosis (> 70%) in one or more major coronary arteries.

### Statistical analysis

Continuous variables are expressed as medians and interquartile ranges (IQR), and categorical data as absolute numbers and percentages. All comparisons were done between 90-day survivors and non-survivors, and between patients with or without STEMI, using the Wilcoxon’s rank sum test for continuous variables and the chi-square test for categorical variables. Survival according to the etiology of CA (STEMI vs. no STEMI) and the revascularization status was assessed using Kaplan–Meier curves, and differences were analyzed through the log-rank test. Variables associated with 90-day mortality were assessed using univariate logistic regression analysis for continuous variables and contingency analysis with Pearson’s test for categorical variables. The analyses were done in the whole population and was repeated separately in STEMI and no STEMI patients. Variables significantly associated with mortality in univariate analysis were introduced in a multivariable regression model. Owing to a limited number of events (death at day 90, *n* = 55), we performed two separate analyses to limit the number of covariables, to avoid biased regression coefficients [12, 13]. The first one included as co-variables age and cardiac arrest variables (shockable rhythm, duration of low flow and no flow), the second included only laboratory variables (lactate, potassium, sodium and glucose). The likelihood ratio test was applied to assess the significance of the variables. For all analyses, a p value < 0.05 was considered statistically significant. Furthermore, given the possibility that plasma potassium could be influenced by variations of arterial pH, we performed bivariate analyses to determine possible linear associations between plasma potassium and arterial pH, and we also assessed possible correlations between arterial pH and plasma sodium and lactate. The JMP^®^ statistical software, version 15, was used for all the analyses.

## Results

**Fig. 1:**
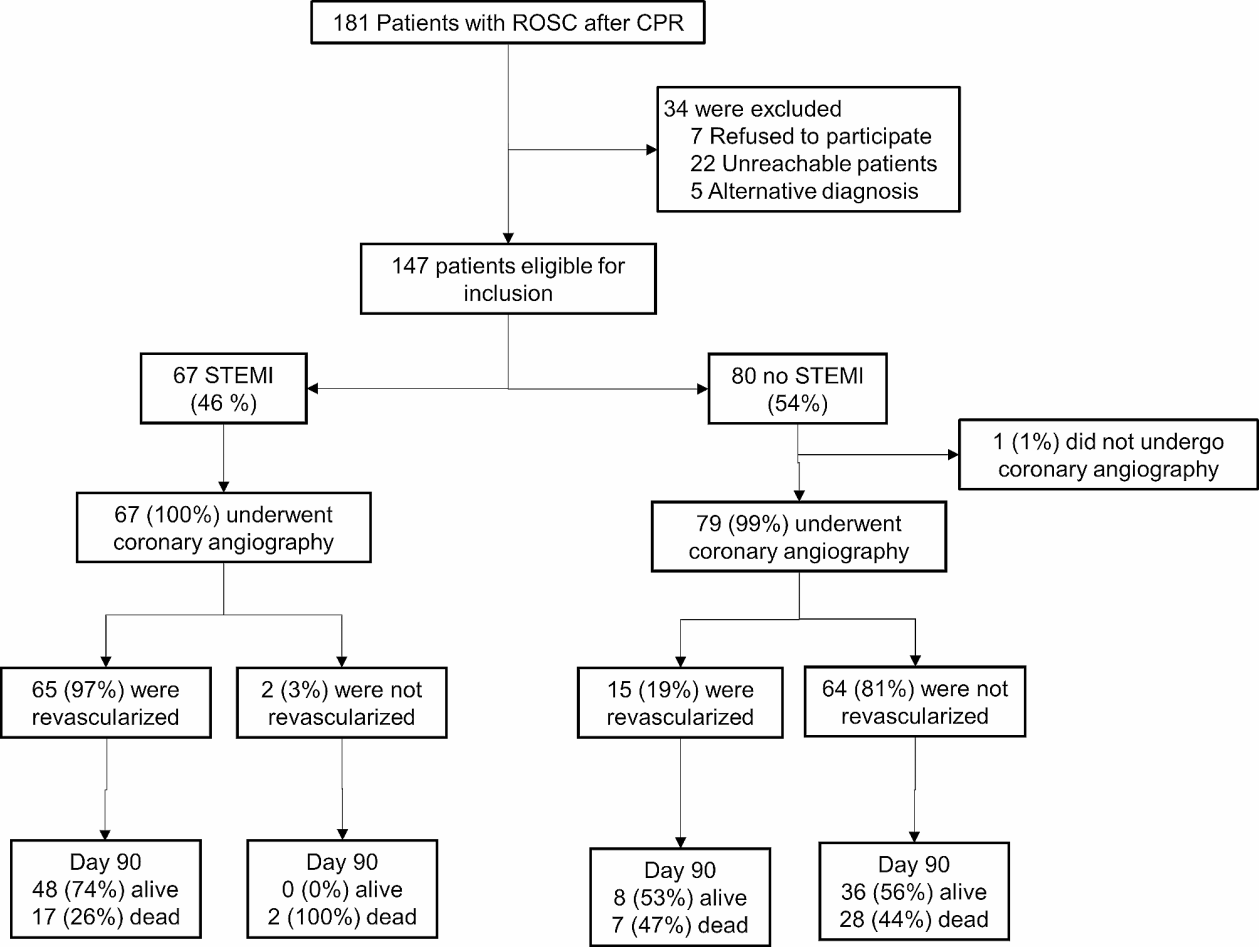
Study flowchart

The flow chart of the study is depicted in Fig. 1. A total of 181 patients were included in the “ROSC pathway”. After exclusion of 34 patients (7 refused to participate, 22 were unreachable for consent and 5 patients had syncope as an alternative diagnosis to CA), a cohort of 147 patients undergoing immediate coronary angiography was analyzed, including 67 STEMI patients and 80 no STEMI patients. In 1 no STEMI patient, angiography was not performed due to a suspected non cardiac etiology of CA (hypoxic cardiac arrest). Coronary revascularization was performed in all but 2 STEMI patients (failed attempt), whereas it was done in 15 no STEMI patients (19%). In 3 patients (1 STEMI and 2 no STEMI), revascularization was performed by emergent coronary artery bypass graft surgery. In no STEMI patients, revascularization was decided by the interventional cardiologist in charge of the patient, only if a suspected culprit coronary occlusion was identified.

Baseline characteristics of the patients are shown in Table 1. In the whole cohort, non-survivors were older and had a more frequent history of diabetes and peripheral arterial disease. The only significant differences between STEMI and no STEMI patients were a greater prevalence of diabetes in no STEMI and a more frequent smoking history in STEMI patients.

**Table 1:** Characteristics of patients

Variable	All (*n*=147)	Alive (*n*=92)	Dead (*n*=55)	*p* value	STEMI (*n*=67)	No STEMI (*n*=80)	*p* value
OHCA, n (%)	133 (90)	82 (92)	51 (93)	0.472	60 (90)	73 (91)	0.727
No-flow, min	0 (0-4)	0 (0-2)	5 (0-10)	<0.001*	0 (0-4)	0 (0-5)	0.555
Low-flow, min	13 (6-25)	10 (5-20)	19 (11-30)	<0.001*	11 (4-23)	15 (7-25)	0.212
Time to ROSC, min	15 (7-25)	10 (5-20)	19 (11-30)	<0.001*	13 (5-29)	18 (10-25)	0.245
Duration on site, min	32 (25-39)	31 (25-36)	33 (26-46)	0.072	32 (27-38)	31 (24-40)	0.825
Arrest witnessed, n (%)	128 (88.3)	87 (95.6)	41 (75.9)	<0.001*	61 (91.0)	67 (85.9)	0.337
AED/Defibrillator, n (%)	44 (30.6)	35 (38.9)	9 (16.7)	0.005*	21 (31.8)	23 (29.5)	0.762
Shockable rhythm, n (%)	117 (79.6)	85 (92.4)	32 (58.2)	<0.001*	60 (89.6)	57 (71.3)	0.006

Continuous variables are presented as medians with interquartile range (IQR). Categorical variables are reported as numbers and percentages. BMI: Body Mass Index; CABG: Coronary Artery Bypass Graft; CAD: Coronary Artery Disease; CVA: Cerebro-Vascular Accident; MI: Myocardial Infarction; PTCA: Percutaneous Transluminal Coronary Angioplasty; STEMI: ST Elevation Myocardial Infarction; No STEMI: Diagnoses other than STEMI

* indicates statistical significant differences

**Fig. 2:**
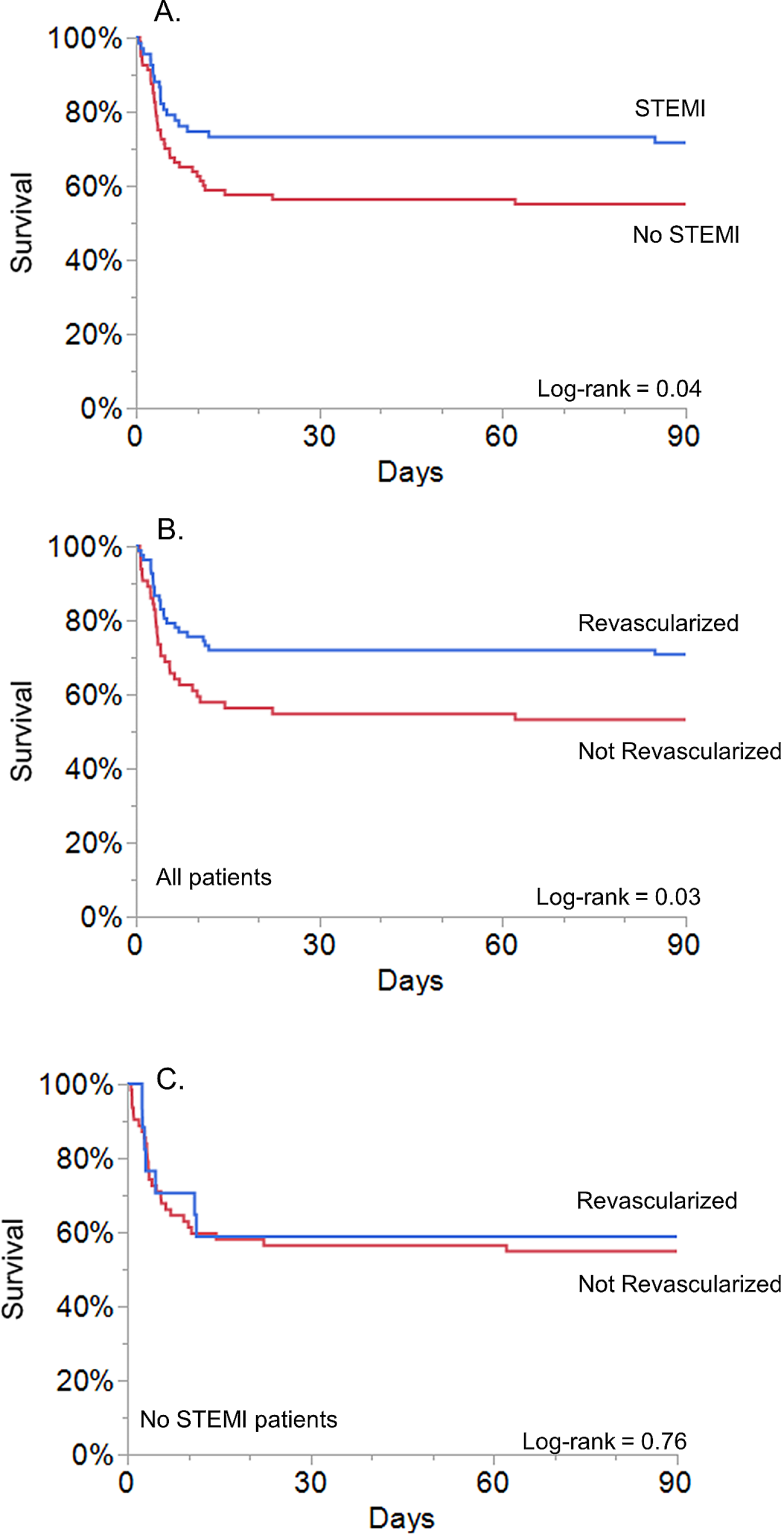
Kaplan-Meier analyses of survival. (**A**) 90-day survival in patients with or without STEMI on initial ECG. (**B**) 90-day survival in revascularized and non-revascularized patients from the whole cohort. (**C**) 90-day survival in revascularized and non-revascularized patients from the No STEMI cohort

The primary endpoint of survival at day 90 occurred in 92/147 (62.5%) patients. Survival was significantly better in STEMI patients (48/67, 72%) than in no STEMI patients (44/80, 55%), as illustrated in the Kaplan-Meier curve depicted in Fig. 2a (*p* = 0.04, log-rank test). In the whole population, 90-day survival was better when emergent coronary revascularization was performed (Fig. 2b, *p* = 0.03, log-rank), whereas in the subgroup of no STEMI patients, survival was not influenced by the revascularization status (Fig. 2c, log-rank = 0.76). The characteristics of cardiac arrest are shown in Table 2. In the whole population, survivors had significantly shorter durations of no flow, low flow and time to ROSC. Witness arrest, the presence of a defibrillator, and a shockable rhythm were more frequently noted in survivors than non-survivors. When comparing STEMI and no STEMI patients, the only significant difference was a more frequent initial shockable rhythm in patients with STEMI (89.6 vs. 71.3%, *p* = 0.006).

**Table 2:** Cardiac arrest characteristics

Variable	All (*n*=147)	Alive (*n*=92)	Dead (*n*=55)	*p* value	STEMI (*n*=67)	No STEMI (*n*=80)	*p* value
OHCA, n (%)	133 (90)	82 (92)	51 (93)	0.472	60 (90)	73 (91)	0.727
No-flow, min	0 (0-4)	0 (0-2)	5 (0-10)	<0.001*	0 (0-4)	0 (0-5)	0.555
Low-flow, min	13 (6-25)	10 (5-20)	19 (11-30)	<0.001*	11 (4-23)	15 (7-25)	0.212
Time to ROSC, min	15 (7-25)	10 (5-20)	19 (11-30)	<0.001*	13 (5-29)	18 (10-25)	0.245
Duration on site, min	32 (25-39)	31 (25-36)	33 (26-46)	0.072	32 (27-38)	31 (24-40)	0.825
Arrest witnessed, n (%)	128 (88.3)	87 (95.6)	41 (75.9)	<0.001*	61 (91.0)	67 (85.9)	0.337
AED/Defibrillator, n (%)	44 (30.6)	35 (38.9)	9 (16.7)	0.005*	21 (31.8)	23 (29.5)	0.762
Shockable rhythm, n (%)	117 (79.6)	85 (92.4)	32 (58.2)	<0.001*	60 (89.6)	57 (71.3)	0.006*

Continuous variables are presented as medians with interquartile range (IQR). Categorical variables are reported as numbers and percentages. AED: automated external defibrillator; ROSC: return of spontaneous circulation

* indicates statistical significant differences

Laboratory variables on admission are presented in Table 3. Survivors displayed significantly higher values of arterial pH, and lower values of plasma lactate, creatinine, urea, potassium, glucose and CKMB, whereas they had higher values of plasma sodium than non-survivors. Comparison between STEMI and no STEMI patients only revealed significantly higher values of plasma CKMB and HS TnT in STEMI patients. The characteristics of admission ECG and coronary angiography in STEMI and no STEMI patients are shown in Table 4. STEMI patients had all ST segment elevation, except from 4 patients presenting with posterior MI on initial ECG. In No STEMI patients, 14 patients (18%) had no ECG modifications, 3 patients (4%) had ST segment elevation (these 3 patients were included in the No STEMI population, given the absence of any coronary disease in one patient, the absence of acute coronary occlusion in another patient, and the absence of MI, as indicated by the lack of any increase in HS TnT, in another patient), and 63 patients (79%) had other ECG modifications. Coronary artery disease with a culprit lesion was present in all STEMI patients, who all underwent emergent revascularization, except in 2 patients (failed attempt). Coronary disease was noted in 40 patients with No STEMI (50%), with 14 patients (18%) displaying a probable culprit lesion leading to emergent revascularization. One additional No STEMI patient underwent revascularization on a non-culprit lesion (chronic coronary occlusion).

**Table 3:** Laboratory values at admission

Variable	All (*n*=147)	Alive (*n*=92)	Dead (*n*=55)	*p* value	STEMI (*n*=67)	No STEMI (*n*=80)	*p* value
pHa	7.19 (7.06-7.31)	7.26 (7.16-7.34)	7.10 (7.02-7.17)	<0.001*	7.20 (7.10-7.34)	7.17 (7.06-7.28)	0.195
Lactate, mmol/L	4.8 (2.3-7.1)	3.6 (1.8-5.8)	6.7 (4.7-9.4)	<0.001*	4.3 (2.13-7.19)	5.0 (3.2-6.91)	0.29
Creatinin, µmol/L	114 (94-134)	105 (87-122)	127 (113-155)	<0.001*	106 (94-126)	119 (95-138)	0.075
Potassium, mmol/L	4.0 (3.6-4.6)	3.9 (3.6-4.2)	4.4 (3.7-5.1)	0.006*	4.0 (3.6-4.5)	4.1 (3.6-4.9)	0.26
Sodium, mmol/L	140 (138-142)	141 (139-142)	140 (137-142)	0.018*	140 (138-142)	141 (138-142)	0.513
BUN, mmol/L	7.0 (5.5-9.2)	6.2 (5.2-8.5)	8.3 (5.9-11.6)	0.003*	6.8 (5.1-9.0)	7.2 (5.7-9.4)	0.212
HbA1c, %	5.6 (5.4-6.1)	5.6 (5.4-6.0)	5.9 (5.3-6.2)	0.481	5.6 (5.4-5.9)	5.6 (5.4-6.2)	0.443
Glucose, mmol/L	12.0 (9.3-15.3)	10.9 (8.7-14.0)	14.1 (10.7-17.3)	<0.001*	12.2 (9.6-16.8)	11.7 (9.0-14.7)	0.23
HS TnT, nmol/L	173 (61-667)	164 (49-644)	174 (81-717)	0.342	372 (89-1727)	127 (15-305)	<0.001*
CK, UI/L	221 (135-388)	214 (122-388)	230 (146-389)	0.495	259 (153-499)	203 (128-362)	0.058
CK-MB, UI/L	85 (54-175)	72 (46-176)	97 (72-175)	0.008*	104 (57-242)	75 (52-130)	0.005*
WBC, G/L	13.7 (9.5-13.3)	13.3 (9.0-17.8)	14.0 (10.2-19.2)	0.342	14.2 (11.0-19.0)	13.5 (8.5-17.6)	0.098

Continuous variables are presented as medians with interquartile range (IQR). BUN: blood urea nitrogen, HbA1c: glycosylated hemoglobin, CK: creatinine kinase, WBC: white blood cell

* indicates statistical significant differences

**Table 4:** ECG and angiography characteristics

Variable	STEMI (*n*=67)	No STEMI (*n*=80)
ST elevation, n (%)	63 (94)	3 (4)
Other ECG modifications, n (%)	4 (6)	63 (79)
No ECG Modification, n (%)	0 (0)	14 (18)
Coronaropathy, n (%)	67 (100)	40 (50)
Culprit lesion		
LAD, n (%)	27 (40)	4 (5)
LCA, n (%)	1 (1)	1 (1)
Cx, n (%)	11 (16)	2 (2) *
RCA, n (%)	25 (37)	3 (4)
Coronary graft, n (%)	2 (3)	0 (0)
Ramus intermedius, n (%)	0 (0)	1 (1)
Undefined (multivessel), n (%)	1 (1)	3 (4)
Revascularization, n (%)	65 (97)	15 (19)

LAD: Left Anterior Descending; LCA: Left Coronary Artery; Cx: Circumflex Artery; RCA: Right Coronary Artery; Other ECG modifications: T wave inversion; ST segment depression; Left Bundle Branch Block. The 4 STEMI patients without ST elevation had posterior MI. The 3 patients without STEMI and ST elevation had no coronary lesion (1 patient), no MI (1 patient), chronic RCA occlusion (1 patient). * One patient without STEMI had revascularization on a non-culprit chronically occluded circumflex artery

The probable causes of cardiac arrest in the population of patients without STEMI (No STEMI) are shown in Supplementary Table 1. A primary cardiac cause was diagnosed in 57 patients, including 41 patients with an underlying ischemic disease (40 with coronary artery disease, and 1 with coronary spasm and no underlying coronary artery disease), and 16 patients without underlying ischemia. Among patients with an ischemic origin, emergent revascularization was performed in 15 patients (14 patients with a culprit acute coronary occlusion, and 1 patient with a chronic occlusion). In 23 patients, a primary non-cardiac cause of the cardiac arrest was considered.

In univariate analyses (Table 5), a diagnosis of STEMI and a positive revascularization status were associated with a reduced 90-day mortality in the whole cohort. In contrast, there was no significant association of revascularization with mortality in the cohort of no STEMI patients. Demographic data associated with greater mortality included an older age, which was only noted in no STEMI patient, as well as a history of chronic arteriopathy and diabetes, the latter being observed only in STEMI, but not in no STEMI patients. With respect to the characteristics of cardiac arrest, a non-shockable rhythm, the absence of witness and longer durations of no flow and low flow were all associated with lower 90-day survival in each individual cohort, whereas the presence of a defibrillator was only positively correlated with survival in STEMI patients. Admission laboratory values showing association with higher 90-day mortality in the total population included higher plasma lactate, glucose and potassium, as well as lower plasma sodium, but the role of high potassium and low sodium was only noted in no STEMI patients when assessing the two cohorts separately. Multivariable analyses (Table 6) indicated that lower age, a shockable rhythm, and shorter no flow and low flow times were independently associated with 90-day survival both in the whole cohort and in the 2 separate cohorts of STEMI and no STEMI patients. With respect to metabolic variables, lower admission lactate and potassium were associated with survival in the whole cohort. In STEMI patients, only lactate was associated with survival, whereas in no STEMI patients, lower potassium and lactate, as well as higher sodium showed significant association with survival. We performed additional analyses incorporating the revascularization status as a co-variable in No STEMI patients. As indicated in Supplementary Table 2, revascularization did not impact 90-day mortality in the tested models.

**Table 5:** Factors associated with 90-day mortality: univariate analysis

	All patients		STEMI		No STEMI	
Variable	OR [95% CI]	*p* value	OR [95% CI]	*p* value	OR [95% CI]	*p* value
STEMI	0.48 [0.24-0.96]	0.037	ND		ND	
Revascularization	0.50 [0.25-0.98]	0.042	ND		1.09 [0.35-3.34]	0.886
Age	1.06 [1.03-1.09]	<0.001	1.04 [0.99-1.10]	0.073	1.06 [1.02-1.11]	0.002
Smoking history	0.69 [0.34-1.35]	0.278	0.52 [0.17-1.57]	0.248	1.03 [0.41-2.60]	0.943
Male Gender	0.75 [0.33-1.77]	0.511	0.54 [0.13-2.16]	0.381	1.03 [0.36-2.96]	0.381
High BP history	1.27 [0.64-2.54]	0.492	1.69 [0.55-5.18]	0.359	1.03 [0.42-2.52]	0.356
Previous CAD	1.12 [0.52-2.37]	0.776	1.81 [0.51-6.43]	0.362	0.73 [0.28-1.90]	0.519
Diabetes	2.79 [1.24-6.37]	0.013	6.25 [1.30-29.9]	0.022	1.63 [0.61-4.35]	0.325
Chronic arteriopathy	2.82 [1.06-7.79]	0.038	2.00 [0.30-13.13]	0.47	2.70 [0.81-8.96]	0.105
CA witnessed	0.15 [0.04-0.44]	<0.001	0.16 [0.03-0.98]	0.048	0.14 [0.03-0.70]	0.017
Defibrillator	0.31 [0.13-0.70]	0.004	0.17 [0.04-0.84]	0.029	0.42 [0.15-1.19]	0.102
No flow time	1.30 [1.17-1.48]	<0.001	1.37 [1.14-1.65]	<0.001	1.26 [1.08-1.46]	<0.001
Low flow time	1.06 [1.03-1.09]	<0.001	1.06 [1.01-1.10]	0.004	1.06 [1.00-1.11]	0.005
Shockable Rhythm	0.11 [0.04-0.28]	<0.001	0.12 [0.02-0.69]	0.019	0.13 [0.04-0.39]	<0.001
Glucose	1.10 [1.03-1.18]	0.003	1.12 [1.02-1.23]	0.012	1.11 [1.00-1.23]	0.029
Lactate	1.32 [1.17-1.50]	<0.001	1.37 [1.14-1.65]	<0.001	1.28 [1.08-1.51]	0.002
Potassium	1.92 [1.28-3.01]	0.001	1.03 [0.52-2.06]	0.925	2.70 [1.47-4.95]	<0.001
Sodium	0.87 [0.79-0.95]	0.001	0.94 [0.83-1.07]	0.355	0.80 [0.69-0.92]	<0.001
Troponin T HS	1.00 [1.00-1.00]	0.219	1.00 [0.99-1.00]	0.217	1.00 [0.99-1.00]	0.083
Leukocytes	1.04 [0.99-1.10]	0.161	1.08 [0.98-1.17]	0.141	1.03 [0.97-1.10]	0.339

**Table 6:** Factors associated with 90-day mortality: multivariable analysis

	All patients		STEMI		No STEMI	
Variable	OR [95% CI]	*p* value	OR [95% CI]	*p* value	OR [95% CI]	*p* value
Age	1.12 [1.06-1.19]	<0.001	1.12 [1.01-1.24]	0.008	1.13 [1.05-1.21]	<0.001
Shockable rhythm	0.03 [0.01-0.16]	<0.001	0.01 [0.00-0.33]	0.001	0.04 [0.00-0.32]	<0.001
No flow	1.45 [1.22-1.72]	<0.001	1.61 [1.19-2.20]	<0.001	1.37 [1.11-1.68]	<0.001
Low flow	1.09 [1.03-1.14]	<0.001	1.09 [1.01-1.18]	0.014	1.10 [1.00-1.20]	0.006
Glucose	0.99 [0.91-1.08]	0.888	1.00 [0.88-1.15]	0.937	0.97 [0.85-1.11]	0.668
Potassium	1.70 [1.06-2.73]	0.023	0.97 [0.44-2.13]	0.938	2.24 [1.17-4.25]	0.011
Sodium	0.91 [0.82-1.02]	0.081	0.96 [0.83-1.10]	0.53	0.84 [0.71-0.99]	0.033
Lactate	1.30 [1.12-1.51]	<0.001	1.35 [1.06-1.72]	0.008	1.24 [1.01-1.52]	0.034

The results of bivariate analyses testing for possible correlations between arterial pH, plasma lactate, potassium and sodium are shown in Supplementary Figure S1. There was a strong negative correlation between arterial pH and plasma lactate (R^2^ = 0.5277, Fig. S1 A). In contrast, there was no linear correlation between arterial pH and plasma potassium (R^2^ = 0.0383, Fig. S1B) and sodium (R^2^ = 0.0176, Fig. S1C). Also, there was no correlation between plasma lactate and potassium (R^2^ = 0.0069, Fig. S1D) or sodium (R^2^ = 0.0292, Fig. S1E). In addition, we addressed the independent predictive value of elevated potassium on 90-day mortality in a multivariable analysis including arterial pH, plasma lactate and plasma potassium as explanatory co-variables, both in the whole population (*n* = 147) and in the population of No STEMI patients (*n* = 80). As shown in Supplementary Figure S2 (forest plot graphs), plasma potassium and arterial pH (whole population), and plasma potassium only (No STEMI patients) were the variables significantly associated with 90-day mortality in this analysis. Owing to the significant negative correlation between arterial pH and plasma lactate, we performed additional multivariable analyses incorporating plasma potassium with either arterial pH or plasma lactate as explanatory covariables. As presented in Supplementary Table 3, these analyses showed that arterial pH and plasma K^+^ (Supplementary Table 3A), as well as plasma lactate and plasma K^+^ (Supplementary Table 3B), were significantly associated with 90-day mortality both in the whole population and in the population of No STEMI patients.

## Discussion

The main results of our study indicate that, in patients successfully resuscitated from cardiac arrest of presumed cardiac etiology and undergoing immediate coronary angiography, there is no survival advantage of emergent revascularization in those patients presenting without ST-segment elevation on admission ECG.

The benefit of emergent coronary angiography and revascularization after resuscitated cardiac arrest in patients presenting with STEMI on initial ECG has been clearly demonstrated [3, 14, 15]. In this setting, immediate PCI reduces the risk of recurrent life-threatening arrhythmias, preserves ventricular function, may help prevent the long-term development of heart failure [1] and has been associated with short and medium-term survival reaching 55–78% and 54–62%, respectively [16–18]. Our results indicating a 90-day survival of 74% in STEMI patients resuscitated from CA and undergoing immediate PCI are in perfect agreement with these previous findings.

A possible benefit from early revascularization in patients without STEMI on initial ECG had also been suggested in a pioneer study by Spaulding et al. [19]. These authors reported that coronary occlusion was frequent in survivors from CA but was poorly predicted by electrocardiographic findings. Furthermore, successful angioplasty was associated with survival in multivariate analysis [19]. Expanding these observations, Dumas et al. reported an improved survival in post-CA patients treated with immediate coronary angioplasty, regardless of the initial ECG [20]. In a recent study in 221 post-CA patients, Caniato et al. found that very early (< 1 h) coronarography significantly reduced early mortality in patients with no ST-segment elevation [6]. Further studies confirmed the benefits from early PCI in patients without STEMI, as shown by improved survival [21] and functional outcome [5].

At variance with these earlier observations, two large recent randomized controlled trials (RCTs) failed to demonstrate any advantage of early revascularization in the population of CA patients without STEMI. The COACT [9] and TOMAHAWK [1] trials compared immediate versus delayed angiography in post-cardiac arrest patients without STEMI on initial ECG and found no difference in survival between the two strategies. Of note, in contrast to our study, COACT only included patients with a shockable initial rhythm, whereas TOMAHAWK included patients regardless of the initial rhythm. Several smaller RCTs, including DISCO [22], EMERGE [23] and PEARL [24] studies, also reported no benefit from early coronarography in the absence of STEMI after CA, although these trials were underpowered to adequately assess the end-points of survival and neurological outcome.

In the current study, we did not compare early vs. delayed coronary angiography in CA patients without STEMI, as was done in the above mentioned RCTs. In contrast, we compared all resuscitated CA patients with or without STEMI undergoing immediate coronary angiography, and we determined the impact on outcome of early revascularization in those without STEMI. Our findings are consistent with these RCTs, confirming, in a real-world setting, the lack of any significant benefit from early revascularization in post-CA patients without STEMI. 90-day survival was indeed comparable in these patients, whether they underwent or not immediate PCI (53% vs. 56%, *p* = 0.76). Importantly, this lack of benefit was observed despite the existence of a culprit coronary lesion in these revascularized patients. Furthermore, it is noteworthy that a significant coronary disease was diagnosed in 50% of patients in the absence of STEMI, which is close to the proportion of coronaropathy reported in patients with no STEMI in other studies [1, 5, 9], but a majority of these patients did not undergo emergent revascularization in the absence of an identified culprit lesion. These findings suggest that an ischemic origin can be held responsible for the cardiac arrest in a significant proportion of patients without STEMI. However, emergent revascularization in these patients (in the presence of a culprit lesion) does not translate into an improved outcome.

Although such lack of benefit may seem counterintuitive at first glance, it may be explained by several factors. The first one is the type of cardiac rhythm observed at the onset on resuscitation. Patients with no STEMI displayed a non-shockable rhythm significantly more often than STEMI patients, which is recognized as a crucial prognostic determinant in cardiac arrest [25]. Furthermore, although not significant, no STEMI patients tended to be older, which is another major factor of lower survival after cardiac arrest [26]. A third point to consider is the delay of post-resuscitation care inevitably associated with the angiographic procedure [1]. This might notably exacerbate ongoing cellular dysfunction triggered by the hypoxic conditions prevailing during cardiac arrest and the oxidative stress developing upon systemic reperfusion during CPR and ROSC. Such hypoxia and reoxygenation triggers a cascades of events culminating into the post-resuscitation disease [27] or post-cardiac arrest syndrome [28], with components of brain injury, cardiovascular dysfunction and systemic inflammation [28, 29].

We identified two metabolic alterations that were significantly associated with mortality in no STEMI patients (as well as in the whole population), which may support the concept of ongoing early post-arrest syndrome in these patients. These changes included higher plasma potassium and lower plasma sodium at admission. We first verified that the changes in plasma electrolytes were not simply related to variations of arterial pH and lactate, by showing a lack of linear correlation between plasma potassium and sodium with either arterial pH or lactate. Furthermore, elevated plasma K^+^ was independently associated with mortality in multivariable analysis incorporating arterial pH and lactate as co-variables. Such concomitant disturbance of plasma Na^+^ and K^+^ is highly suggestive of an impaired activity of the cellular Na^+^/K^+^ ATPase, which is known to develop within minutes of hypoxia as an adaptive mechanism allowing to lower cellular ATP consumption [30]. This downregulation of Na^+^/K^+^ ATPase results both from the endocytosis of its α subunit from the plasma membrane into intracellular pools [31], and from its ubiquitin-dependent degradation, triggered by hypoxia-generated mitochondrial reactive oxygen species [30]. Impaired Na^+^/K^+^ ATPase activity has been notably associated with myocardial [32] and brain [33] dysfunction, two key features of the post-CA syndrome. Our findings therefore suggest that the dysfunction of NA^+^/K^+^ ATPase in response to hypoxia and oxidative stress may be more severe in non-surviving patients without STEMI. This suggests that therapies aiming to rapidly improve tissue oxygen delivery and restore metabolic homeostasis could be more beneficial than emergent revascularization in these patients.

Our study has several limitations. The first one relies in its retrospective design and the relatively limited sample size. Second, our results may be criticized for a lack of novelty, since recent RCTs have shown no benefit from emergent coronary angiography in patients with no ST segment elevation after cardiac arrest. However, it must be underscored that our study was performed in an unselected population, reflecting a real-world clinical context. Furthermore, we identified some metabolic disturbances that were associated with increased mortality, which should deserve further investigations in future studies. Third, we evaluated 90-day mortality, and we may therefore not conclude on longer-term survival and cardiovascular events. Fourth, we did not evaluate neurological outcome, but it is noteworthy that most patients were discharged home after hospitalization, suggesting overall good neurological recovery.

In conclusion, we found that, in a population of post-cardiac arrest patients from presumed cardiac origin undergoing immediate coronary angiography, such procedure did not improve 90-day survival in patients without STEMI on initial ECG. Furthermore, non-surviving patients without STEMI displayed subtle early metabolic disturbances, suggesting that prompt intensive care management might be more useful than immediate coronary angiography in these patients.

### Electronic supplementary material

Below is the link to the electronic supplementary material.

Supplementary Material 1

Supplementary Material 2

Supplementary Material 3

Supplementary Material 4

Supplementary Material 5

Supplementary Material 6

## Data Availability

All data generated or analyzed during this study are included in this article.

## Abbreviations

ACS: Acute Coronary Syndrome
AED: Automated external defibrillator
BMI: Body mass index
BP: Blood pressure
BUN: blood urea nitrogen
CA: Cardiac arrest
CABG: Coronary artery bypass grafting
CAD: Coronary artery disease
CHF: Chronic heart failure
CK: Creatine kinase
CKMB: creatine kinase, MB fraction
CPR: Cardiopulmonary resuscitation
Cx: Circumflex artery
ECG: Electrocardiogram
HbA1c: Glycosylated hemoglobin
HS TnT: High sensitivity troponin T
IHCA: In-hospital cardiac arrest
IQR: Interquartile range
LAD: Left anterior descending
LCA: Left coronary artery
MI: Myocardial infarction
OHCA: Out-of-hospital cardiac arrest
PCI: Primary coronary intervention
PTCA: Percutaneous transluminal coronary angioplasty
RCA: Right coronary artery
RCT: Randomized controlled trial
ROSC: Return of spontaneous circulation
STEMI: ST-elevation myocardial infarction
WBC: white blood cell
