# Supplementary figures and images for: Outcomes and relevance of emergency percutaneous coronary angiography and intervention after resuscitated cardiac arrest: a retrospective study
Source: BMC Cardiovasc Disord. 2024 Aug 13;24:425. doi: 10.1186/s12872-024-04052-1 (PMC11321191; doi:10.1186/s12872-024-04052-1)

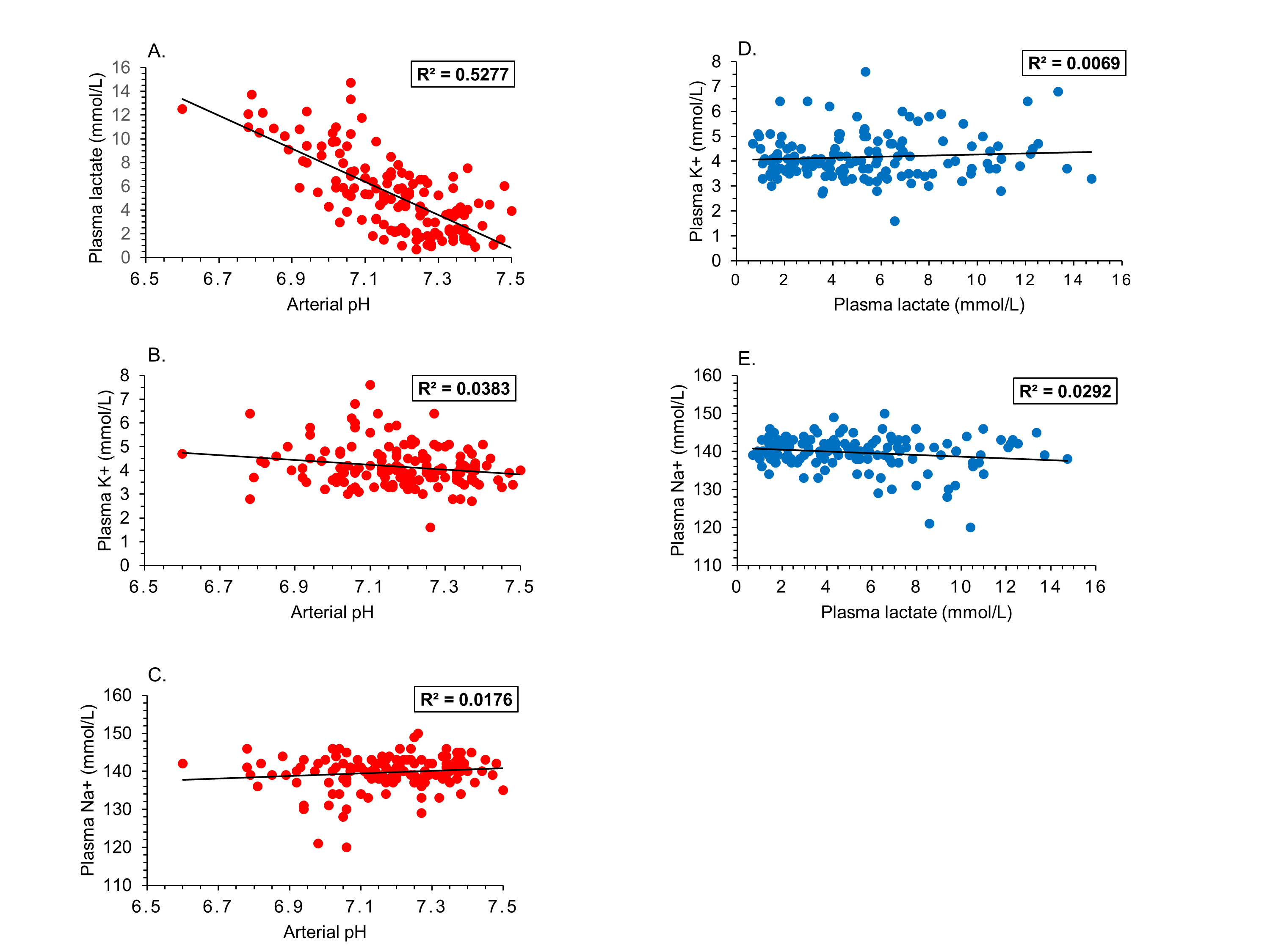

Supplement: Supplementary file 1 — Supplementary Material 1 [file 12872_2024_4052_MOESM1_ESM.tif]

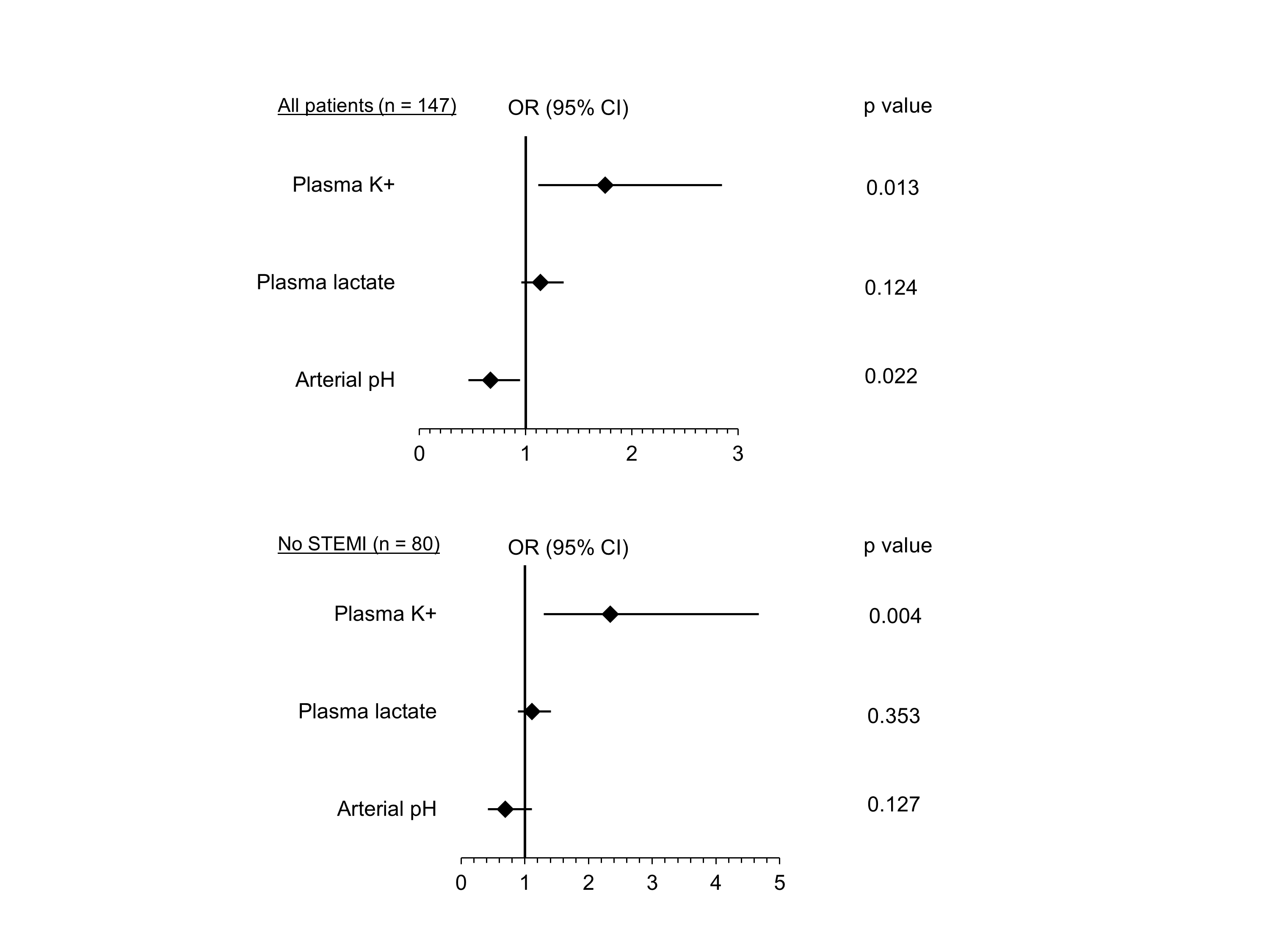

Supplement: Supplementary file 2 — Supplementary Material 2 [file 12872_2024_4052_MOESM2_ESM.tif]
