# Supplementary material for: Outcomes and relevance of emergency percutaneous coronary angiography and intervention after resuscitated cardiac arrest: a retrospective study
Source: BMC Cardiovasc Disord. 2024 Aug 13;24:425. doi: 10.1186/s12872-024-04052-1 (PMC11321191; doi:10.1186/s12872-024-04052-1)
Supplement: Supplementary file 3 — Supplementary Material 3 [file 12872_2024_4052_MOESM3_ESM.docx]

Legends to Supplementary Figures.

Supplementary Figure S1. Correlation studies between arterial pH, plasma lactate, sodium and potassium.

The markers indicate individual data. n =144 (missing data in n = 3 patients)

Supplementary Figure S2. Role of arterial pH, plasma lactate and potassium on 90-day mortality - multivariable analysis (Forest plot graphs)

The influence of arterial pH, plasma lactate and plasma potassium on 90-day mortality was evaluated in a multivariable regression analysis. In the whole population (upper graph), variables significantly associated with 90-day mortality were arterial pH (for each 0.1 increase in pH: OR =0.67, 95% CI [0.46-0.95], p = 0.022) and plasma potassium (for each 1 mmol/L increase, OR = 1.75, 95% CI [1.12-2.85], p = 0.013). In contrast, L-lactate was not independently associated with 90-day mortality (for each 1 mmol/L increase, OR = 1.14, 95% CI [0.96-1.36], p = 0.124). In No STEMI patients (lower graph), plasma K^+^ was significantly associated with mortality (OR = 2.34, 95% CI [1.29-4.68], p = 0.004), whereas both arterial pH (OR =0.69, 95% CI [0.42-1.11], p = 0.127) and plasma lactate (OR = 1.11, 95% CI [0.89-1.41], p = 0.353) were not.
