# Supplementary material for: Outcomes and relevance of emergency percutaneous coronary angiography and intervention after resuscitated cardiac arrest: a retrospective study
Source: BMC Cardiovasc Disord. 2024 Aug 13;24:425. doi: 10.1186/s12872-024-04052-1 (PMC11321191; doi:10.1186/s12872-024-04052-1)
Supplement: Supplementary file 4 — Supplementary Material 4 [file 12872_2024_4052_MOESM4_ESM.docx]

Supplementary Table 1. Etiology of cardiac arrest in No STEMI patients

Etiology of cardiac arrest n (%) CAD, n (%)

*Primary cardiac* 57 (71) 40 (50)

Ischemic heart disease 41 (51) 40 (50)

ACS with a culprit lesion * 14 (18) 14 (18)

ACS without a culprit lesion 7 (9) 7 (9)

Arythmia, myocardial scar ** 16 (20) 16 (20)

Arhythmia, ischemic CHF 1 (1) 1 (1)

Pulmonary edema, ischemic CHF 2 (2.5) 2 (2.5)

Coronary spasm 1 (1) 0 (0)

Non-ischemic heart disease 16 (20) 0

Arhythmia, non-ischemic CHF 4 (5) 0

Long QT syndrome 3 (4) 0

Takotsubo syndrome 3 (4) 0

Malignant mitral prolapse 3 (4) 0

Atrioventricular block 3 (4) 0

*Primary non-cardiac* 23 (29) 0

Hypoxia 4 (5) 0

Pulmonary Embolism 1 (1) 0

Undetermined 18 (23) 0

*All patients in this category underwent emergent revascularization. ** One patient in this category underwent emergent revascularization on a chronically occluded coronary artery.

Abreviations: ACS: Acute Coronary Syndrome; CAD: Coronary Artery Disease; CHF: Chronic Heart Failure.
