# Supplementary material for: Outcomes and relevance of emergency percutaneous coronary angiography and intervention after resuscitated cardiac arrest: a retrospective study
Source: BMC Cardiovasc Disord. 2024 Aug 13;24:425. doi: 10.1186/s12872-024-04052-1 (PMC11321191; doi:10.1186/s12872-024-04052-1)

Supplementary Table 2. Impact of revascularization on 90-day mortality in No STEMI patients: multivariable analyses

Variable OR [95% CI] p value

Age 1.13 [1.06-1.23] <0.001

Shockable rhythm 0.05 [0.01-0.28] 0.004

No flow 1.39 [1.15-1.80] <0.001

Low flow 1.10 [1.02-1.22] <0.001

Revascularization 2.32 [0.38-14.78] 0.356

Glucose 0.97 [0.85-1.11] 0.667

Potassium 2.23 [1.21-4.47] 0.009

Sodium 0.84 [0.69-0.98] 0.022

Lactate 1.24 [1.02-1.55] 0.027

Revascularization 2.29 [0.62-8.79] 0.214


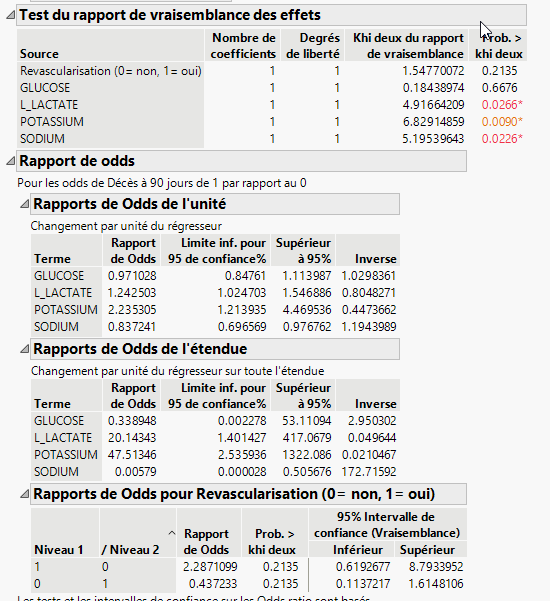


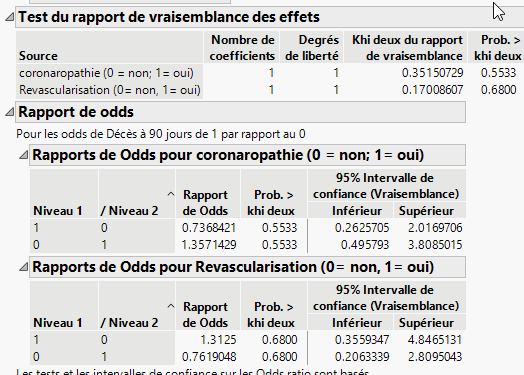


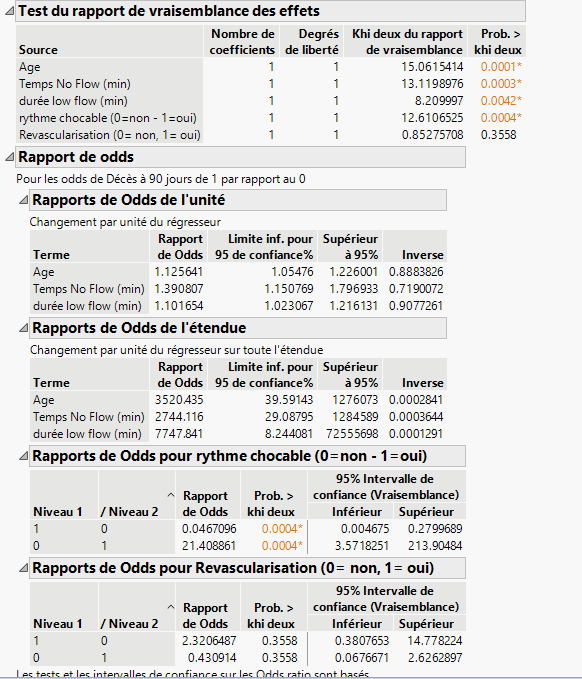

Supplement: Supplementary file 5 — Supplementary Material 5 [file 12872_2024_4052_MOESM5_ESM.docx]
