# Supplementary material for: Outcomes and relevance of emergency percutaneous coronary angiography and intervention after resuscitated cardiac arrest: a retrospective study
Source: BMC Cardiovasc Disord. 2024 Aug 13;24:425. doi: 10.1186/s12872-024-04052-1 (PMC11321191; doi:10.1186/s12872-024-04052-1)
Supplement: Supplementary file 6 — Supplementary Material 6 [file 12872_2024_4052_MOESM6_ESM.docx]

Supplementary Table 3. Impact of pH, potassium and lactate on 90-day mortality: bivariable analyses

All patients No STEMI

Variable OR [95% CI] p value OR [95% CI] p value

Arterial pH 0.55 [0.41-0.70] <0.001 0.59 [0.39-0.85] 0.003

Plasma potassium 1.68 [1.09-2.71] 0.019 2.28 [1.26-4.56] 0.005


Variable OR [95% CI] p value OR [95% CI] p value

Plasma lactate 1.32 [1.17-1.51] <0.001 1.25 [1.06-1.51] 0.006

Plasma potassium 1.92 [1.24-3.09] 0.003 2.55 [1.44-5.02] <0.001

OR and 95% CI were calculated per 0.1 unit change for arterial pH and per 1 mmol/L change for plasma potassium and plasma lactate.
